# Pyoderma Gangrenosum Secondary to Melanotan

**DOI:** 10.7759/cureus.98297

**Published:** 2025-12-02

**Authors:** Daniel Dickson, Su Hlaing Htwe

**Affiliations:** 1 Dermatology, Glasgow Royal Infirmary, Glasgow, GBR

**Keywords:** diagnosis and treatment of pyoderma gangrenosum, drug-induced pyoderma gangrenosum, melanotan, superficial pyoderma gangrenosum, ulcerative pyoderma gangrenosum

## Abstract

Pyoderma gangreonsum (PG) is a rare neutrophilic dermatosis that presents as a rapidly enlarging ulcer. Although the exact aetiology is unclear, up to half of the cases are linked to a systemic inflammatory disorder such as rheumatological conditions, haematology malignancy, or inflammatory bowel disease. A 65-year-old woman reported utilising Melanotan and subsequently developing ulcerated wounds at the injection sites on her abdomen. Clinically, four wounds were noted with an erythematous edge corresponding to the injection sites of melanotan. A diagnosis of PG was obtained due to the wounds’ appearance, progression rate, lack of response to multiple courses of antibiotics, and negative bacteriology, including Panton-Valentine leukocidin *Staphylococcus aureus*. Topical betamethasone, alongside steroid occlusion tape and prednisolone, was commenced with good effect. Prednisolone was slowly titrated, and topical therapy with Dermovate was initiated. The wounds healed with classic cribriform scars after several months of treatment. Drug-induced PG has rarely been documented. However, melanotan-induced PG has never been reported in the literature before. We hope to highlight this case to increase awareness of the side effects of melanotan.

## Introduction

Melanotan is a synthetic analogue of the peptide alpha melanocyte-stimulating hormone, which stimulates the melanocortin type 1 receptor to stimulate melanogenesis and cause tanning [1]. Secondary effects include increased libido and penile erections. Melanotan has been popularised on social media as a beauty product and can be purchased online easily, either as a subcutaneous injection or a nasal spray. The use of it is currently unregulated, and side effects such as priapism and architectural changes of nevi have been reported previously [2].

Typically, pyoderma gangrenosum (PG) is associated with systemic inflammatory disorders such as inflammatory bowel disease, rheumatoid arthritis, and haematological malignancies. Very rarely, it can be drug-induced, and reported culprits include isotretinoin, tyrosine kinase inhibitors, and cocaine [3]. PG can exist as an ulcerative or classic subtype, which begins as small pustules and rapidly ulcerate into painful lesions with violaceous borders, vegetative PG with superficial ulcers lacking a violaceous border, bullous PG with painful, rapidly spreading superficial bullae with inflamed blue grey borders, or peristomal PG in which lesions occur near stoma sites [3,4].

Pyoderma is considered a neutrophilic inflammatory disease. Diagnosis is usually made clinically by the classic appearance and exclusion of other causes. Pathology demonstrates dermal infiltration of mature neutrophils and evidence of necrosis and haemorrhage at the base of the lesions [1]. Management consists of both potent topical steroids, wound care, and, in severe cases, systemic therapy with oral corticosteroids or cyclosporine and mycophenolate mofetil. Case reports exist of tumour necrosis factor-alpha inhibitors such as adalimumab and infliximab being utilised with good effect have also been reported with some efficacy [3].

## Case presentation

A 65-year-old woman presented to the dermatology clinic with an ulcerated lesion and three areas of impending ulceration on her lower abdomen. Two months before seeking medical attention, she had injected melanotan into her abdomen according to the instructions on the website from which she obtained her melanotan injection. The ulcerated areas corresponded to the four injection sites used. Two weeks following the injection of melanotan, the patient noticed small, tender, red papules. Over the following week, these lesions developed into large, painful areas with a necrotic centre, surrounded by a violaceous border. The lesions progressively increased in size, and she developed four distinct ulcers on her abdomen. The patient denied any injury to the area. She had no significant past medical history, particularly no autoimmune or inflammatory conditions. Inflammatory markers were raised with an erythrocyte sedimentation rate of 39 mm/hour; normal C-reactive protein, full blood count, urea and electrolytes, and liver function tests; and a negative antinuclear antibody. Chest X-ray and blood films were normal.

**Figure 1:**
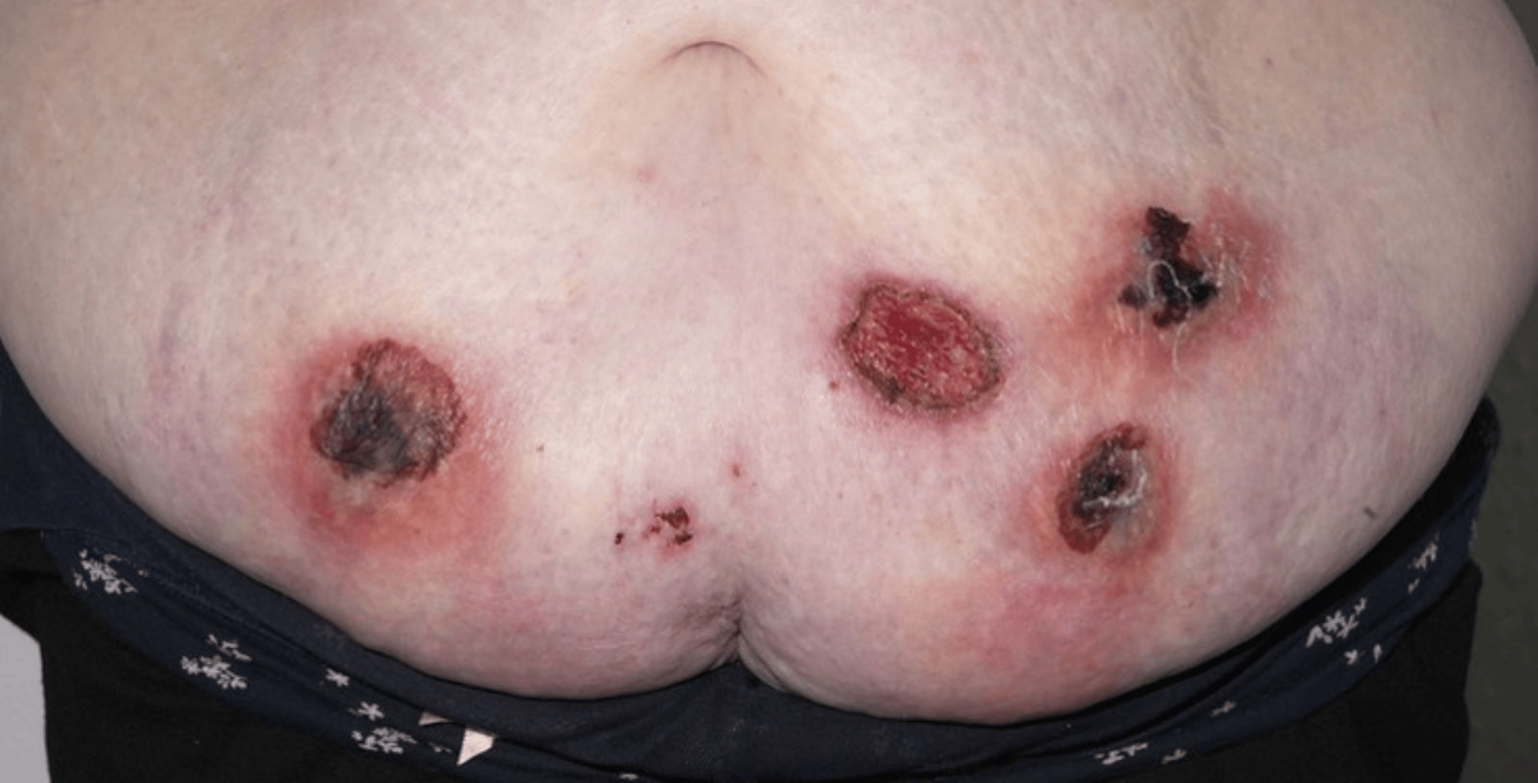
Initial presentation showing an ulcerated violaceous lesion with three impending areas of ulceration.

**Figure 2:**
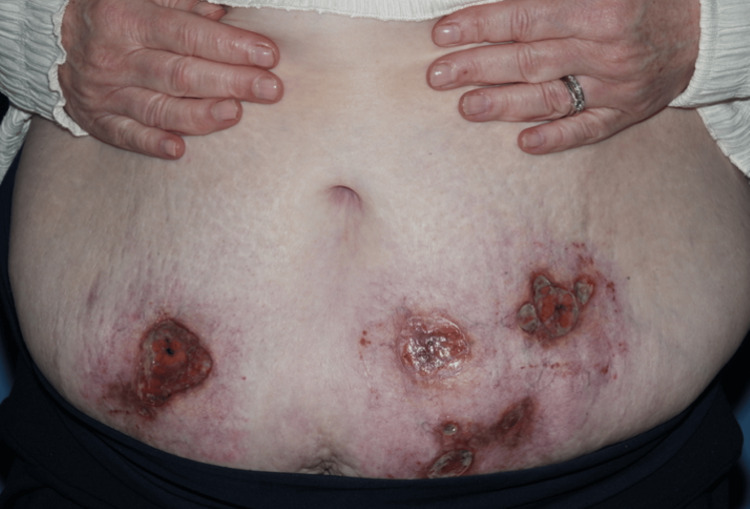
Three weeks after the initial review showing four ulcerated lesions.

**Figure 3:**
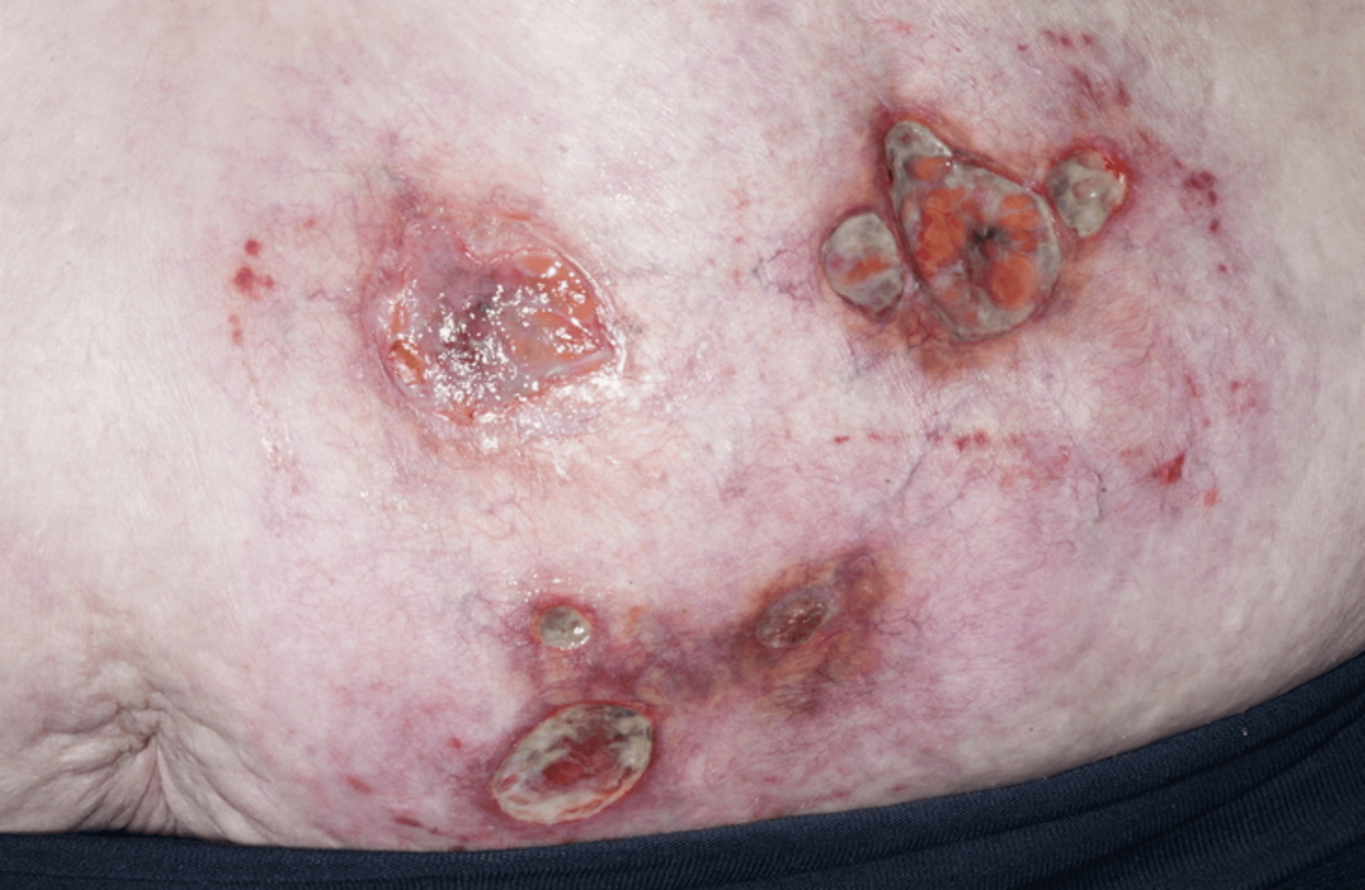
A detailed view of the ulcerated areas.

On examination, an ulcer with a violaceous border and three other areas of impending ulceration were noted. Initial microbiology cultures from her primary care physician were positive for *Staphylococcus aureus*. Flucloxacillin was initiated as per the sensitivity analysis. However, there was no improvement in the patient’s condition. A Panton-Valentine leukocidin *S. aureus* was considered within the differential, but microbiology testing ruled this out. Fludroxycortide tape was recommended to be applied to the abdominal wall, and Betnovate with cloquinol cream to the ulcer borders. Despite this intervention, the patient’s condition continued to worsen. Hence, oral prednisolone was initiated at 30 mg daily. On review two weeks later, three further areas of ulceration were noted with violaceous borders. Figures 1-3 demonstrate the clinical findings.

**Figure 4:**
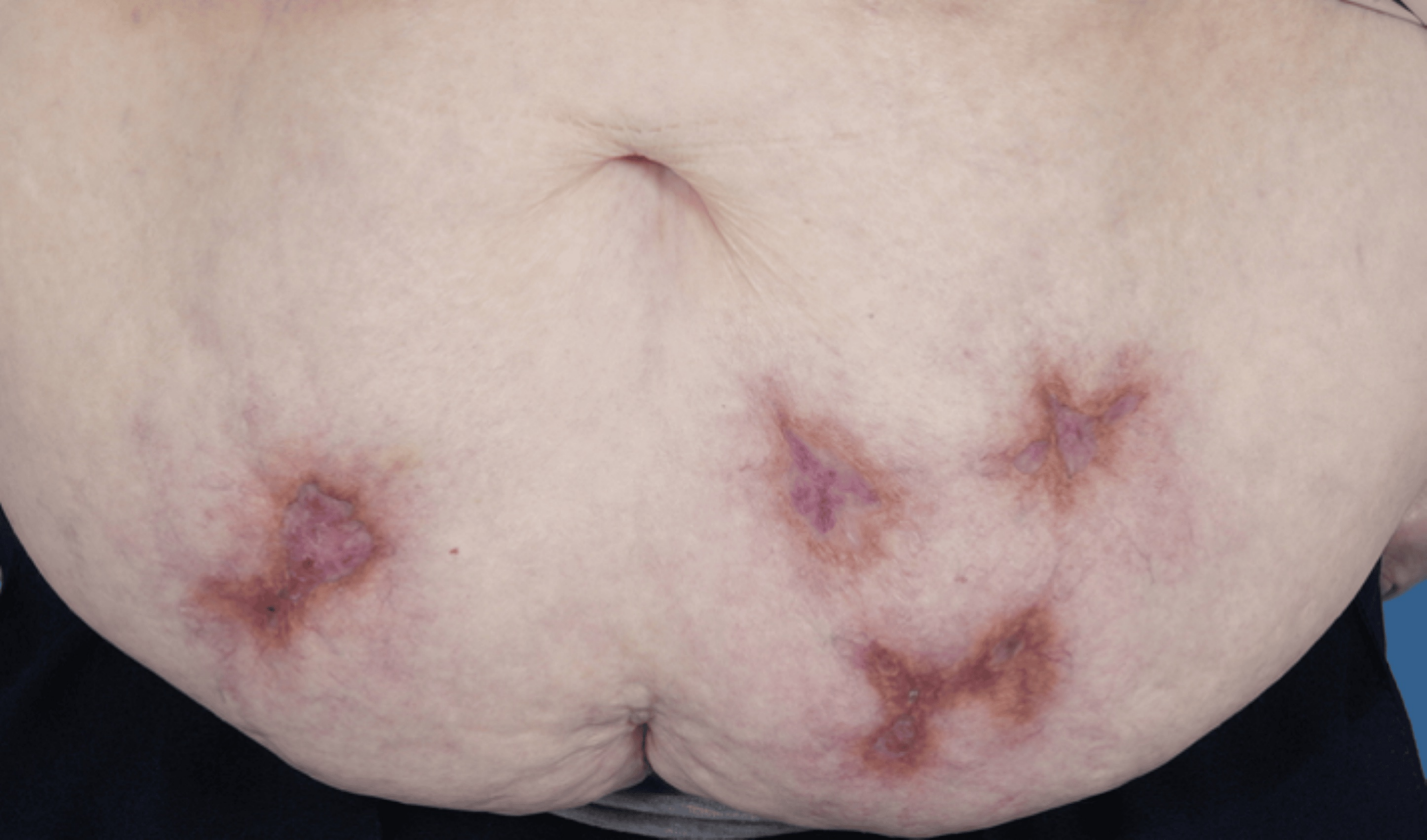
Healed lesions following intensive topical therapy.

The patient was reviewed after three days of oral steroid therapy and, unfortunately, reported that side effects such as anxiety and insomnia were extremely troublesome. Prednisolone therapy was decreased to 20 mg once daily with a slowly weaning dose, with continued bone and gastric protection. The areas of ulceration appeared shallower following the above treatments. Prednisolone was discontinued as per the patient’s request after a slow wean, and clobetasone propionate was recommended to be applied to the ulcerated edges and base. Further systemic treatment options, such as cyclosporin, were discussed; however, our patient elected to continue with topical treatments. On further review, there was evidence of healing and no progression; therefore, topical therapy was felt appropriate and continued. Throughout, wound care was achieved with non-adherent dressings and hydrogel to remove any eschar areas. The PG healed with a classic cribriform scar after several months of topical treatment (Figure 4).

## Discussion

Drug-induced PG is a recognised entity. Particular associations between PG and cocaine laced with levamisole have been reported within the literature [5]. Further, drugs associated with PG include isotretinoin, propylthiouracil, and sunitinib [6]. The mechanisms behind drug-induced PG typically involve activation of neutrophils [7]. Drug release of chemokines promotes migration and activation of neutrophils. Propylthiouracil was found to incite myeloperoxidase antineutrophil cytoplasmic antibodies and IgG antibodies, which, in turn, lead to the activation of neutrophils [7]. Isotretinoin has been shown to increase matrix metalloproteinases-9 (MMP-9), which acts to attract neutrophils to a site of inflammation [7].

Tyrosine kinase inhibitors have also been associated with drug-induced PG, with sunitinib having the greatest number of cases associated with PG. Other tyrosine kinase inhibitors found to be associated include imatinib, ibrutinib, gefitinib, pazopanib, dabrafenib, and trametinib. The pathogenesis of tyrosine kinase inhibitor-induced PG is unclear but is likely due to keratinocyte necrosis and subsequent neutrophilic recruitment [8].

Melanotan is commonly administered via a subcutaneous injection. Interestingly, a case report exists of insulin delivered by subcutaneous injection causing PG [6]. It is theorised that subcutaneous insulin leads to increases in MMP-9 and resultant neutrophilic inflammation [6].

It is possible that melanotan delivered in a subcutaneous fashion has similar MMP-9-inducing effects and promotes neutrophil recruitment and resulting PG. Drug-induced PG is a recognised entity and should be suspected when PG develops temporally to new drug use in the absence of systemic factors such as haematological malignancy or systemic inflammatory conditions.

## Conclusions

This is the first case report of PG secondary to melanotan injections described within the literature. In our patient, PG developed at sites in which melanotan was injected. It is difficult to differentiate whether it was the constituents of the melanotan itself or the trauma of the injection (pathergy) that caused PG in our patient. It is important to recognise that melanotan is not manufactured by legitimate pharmaceutical companies, and, as such, adverse effects are mostly reported through case reports rather than through the Medicines and Healthcare Products Regulatory Agency Yellow Card system. Our case highlights the unusual development of PG at sites of melanotan injection. Physicians should be mindful of unreported adverse effects of melanotan use and counsel patients regarding the dangers of this treatment.
